# Supplementary figures and images for: A vertebra of a small species of Pachycetus from the North Sea and its inner structure and vascularity compared with other basilosaurid vertebrae from the same site
Source: PeerJ. 2024 Jan 25;12:e16541. doi: 10.7717/peerj.16541 (PMC11107809; doi:10.7717/peerj.16541)

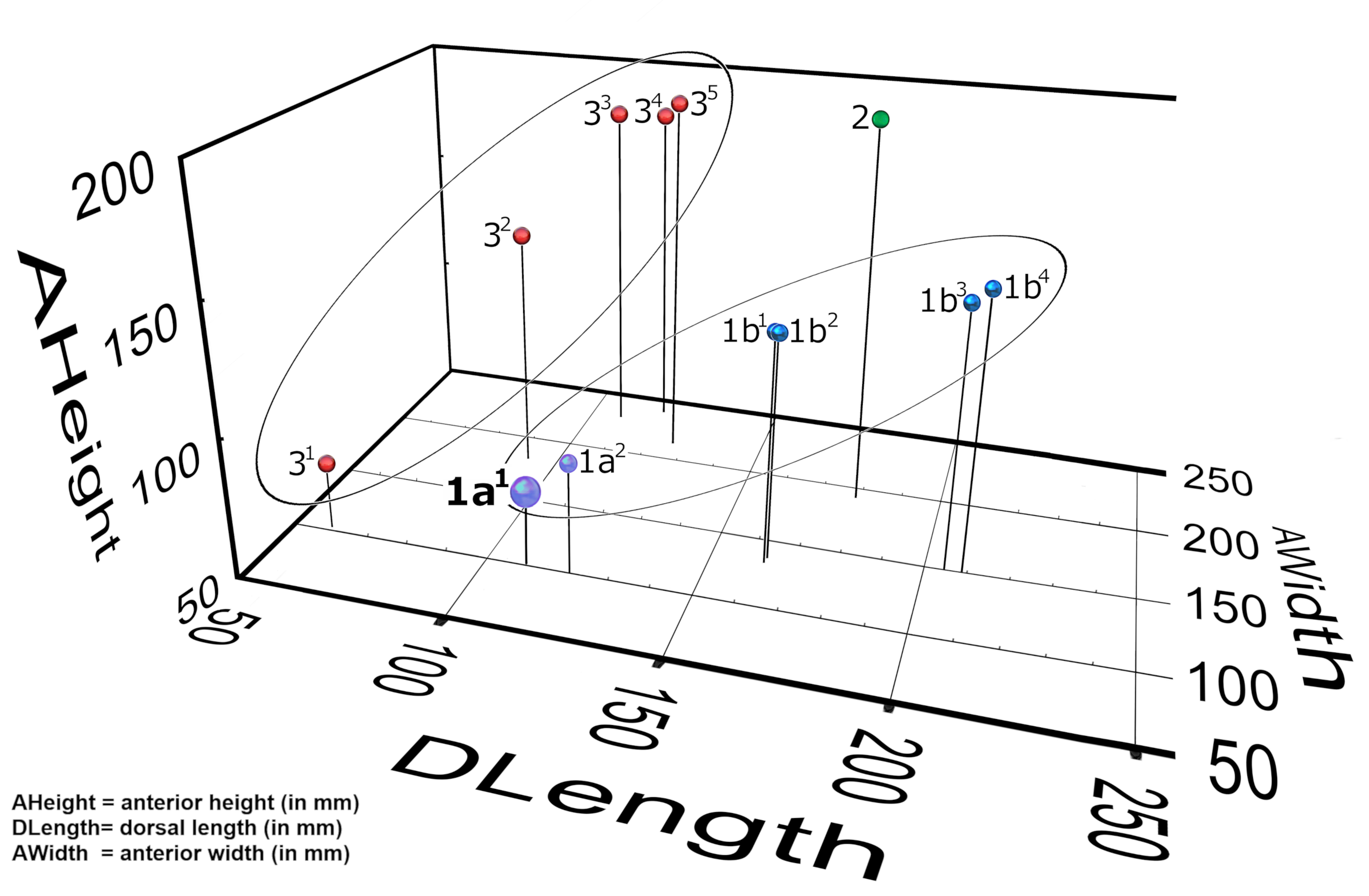

Supplement: Figure S1 — Morphotypes 1, 2 and 3 (with elongated, not-elongated, resp. ‘shortened’ torso vertebrae) from the region of Wielingen (Dutch-Belgian border), and Het Scheur (Belgium). The dorsal length, anterior width and anterior height of the vertebral centra are indicated in a three-dimensional diagram. Vertebrae of Morphotype 1a: blue-coloured spheres 1a1-2 (1a1: NMR-16642; 1a2:NMR-150839), small species of Pachycetus. Vertebrae of Morphotype 1b: blue-coloured spheres 1b1-4 (1b1: NMR-12332; 1b2: NMR-12331; 1b3:NMR-16645; 1b4:NMR-3404), large species of Pachycetus. Vertebra of Morphotype 2: green-coloured sphere 2 (2: NMR-10284), indeterminable basilosaurid. Vertebrae of Morphotype 3: red-coloured spheres 31-5 (31: NMR-160635; 32: NMR-3882; 33: NMR-3403; 34: NMR-3402; 35: NMR-10283), indeterminable basilosaurid. See also Table S1. [file peerj-12-16541-s001.png]

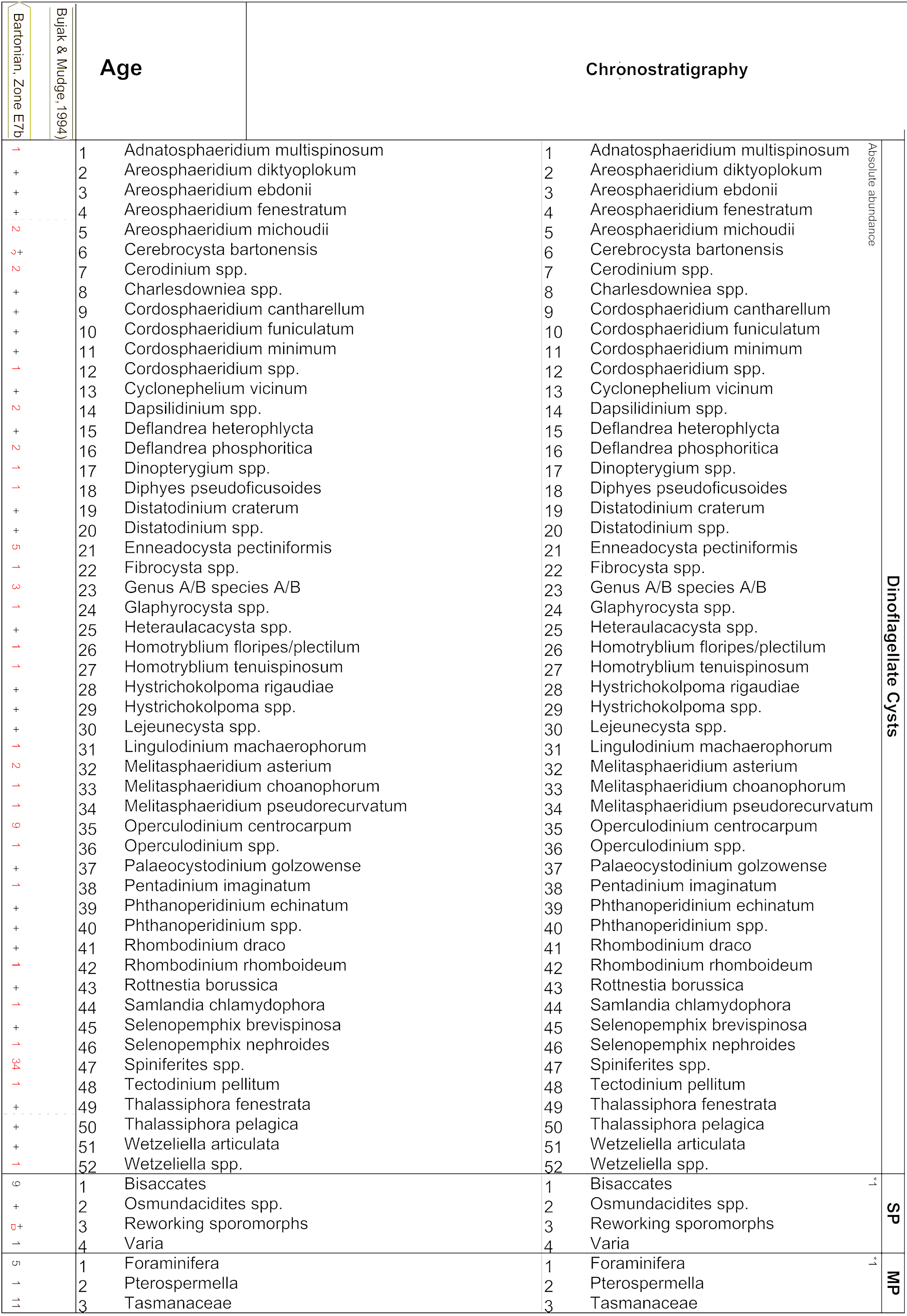

Supplement: Figure S2 — The encountered species are given in the column at the left. Abbreviations: MP Miscellaneous fossils, SP Sporomorphs (bisaccate pollen and spores). [file peerj-12-16541-s002.png]

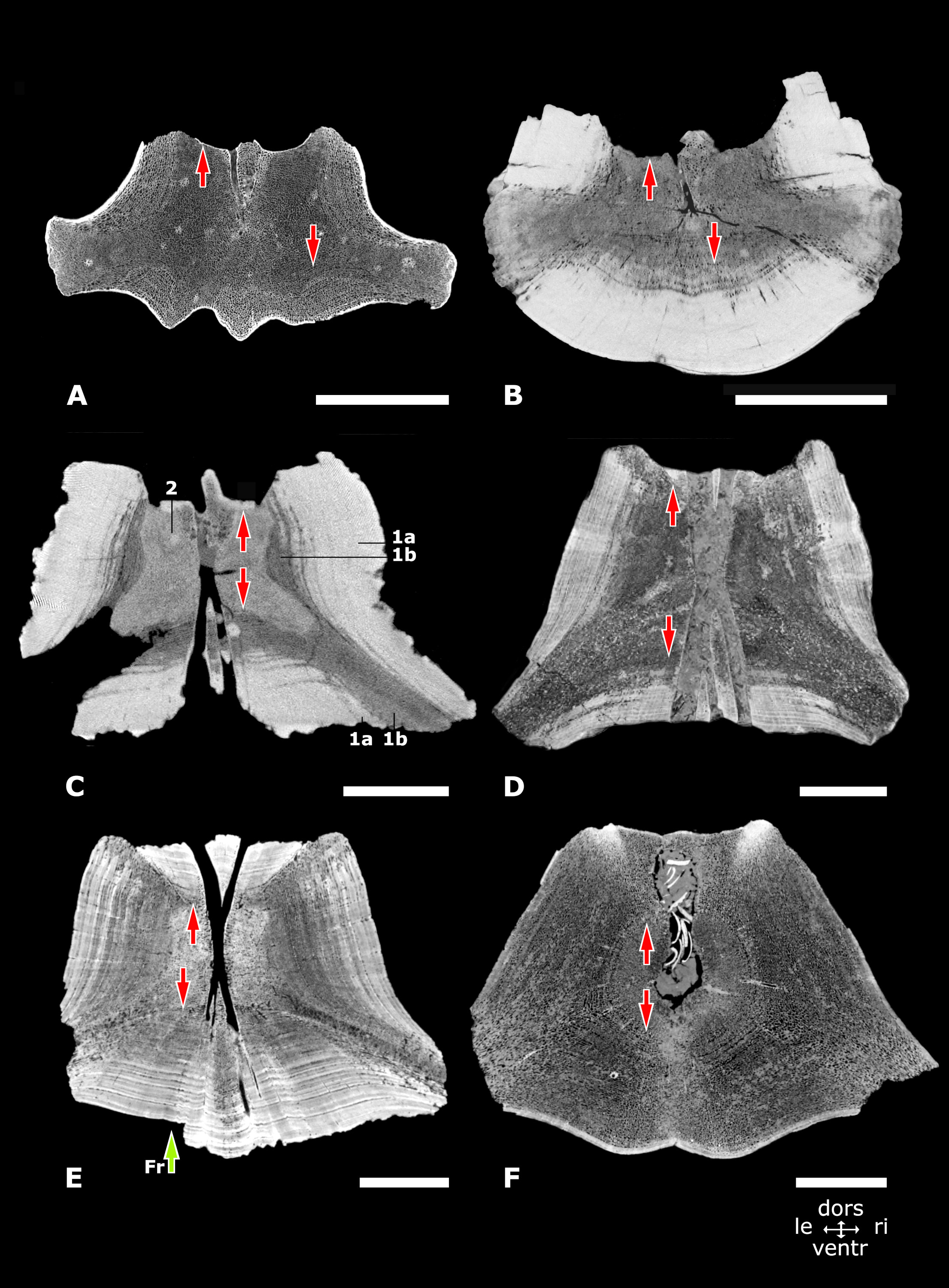

Supplement: Figure S3 — CT-scan images in axial view. from the midpart of vertebral centra in the morphotypes 1, 2 and 3. (A) Morphotype 1a, central-posterior thoracic vertebra, NMR-16642, small species of Pachycetus. (B) Morphotype 1b, central-posterior thoracic vertebra, NMR-12331, large species of Pachycetus. (C) Morphotype 1b, lumbar vertebra, NMR-3404, large species of Pachycetus. (D) Elongated lumbar or caudal vertebral centrum, USNM 510831, Basilosaurus cetoides, modified after (Houssaye et al. (2015): fig. 14a). (E) Morphotype 2, posterior thoracic or lumbar vertebra, NMR-10284, indeterminable basilosaurid. (F) Morphotype 3, caudal vertebra, NMR-10283, indeterminable basilosaurid. Vertical arrows downward point to the ventral cortex; vertical arrows upward point to the dorsal cortex. Green arrow (marked with ‘Fr’) in (E) points to a missing part of the cortex, broken along a less compact layer. See Numbers in (D): 1a, compact outer layers of the cortex; 1b, spongious-like inner layers of the cortex; 2, spongious bone at the midpart of the vertebral centrum; compare with Fig. 5B. also with Suppl. Table S1. Scale bars are 50 mm [file peerj-12-16541-s003.jpg]

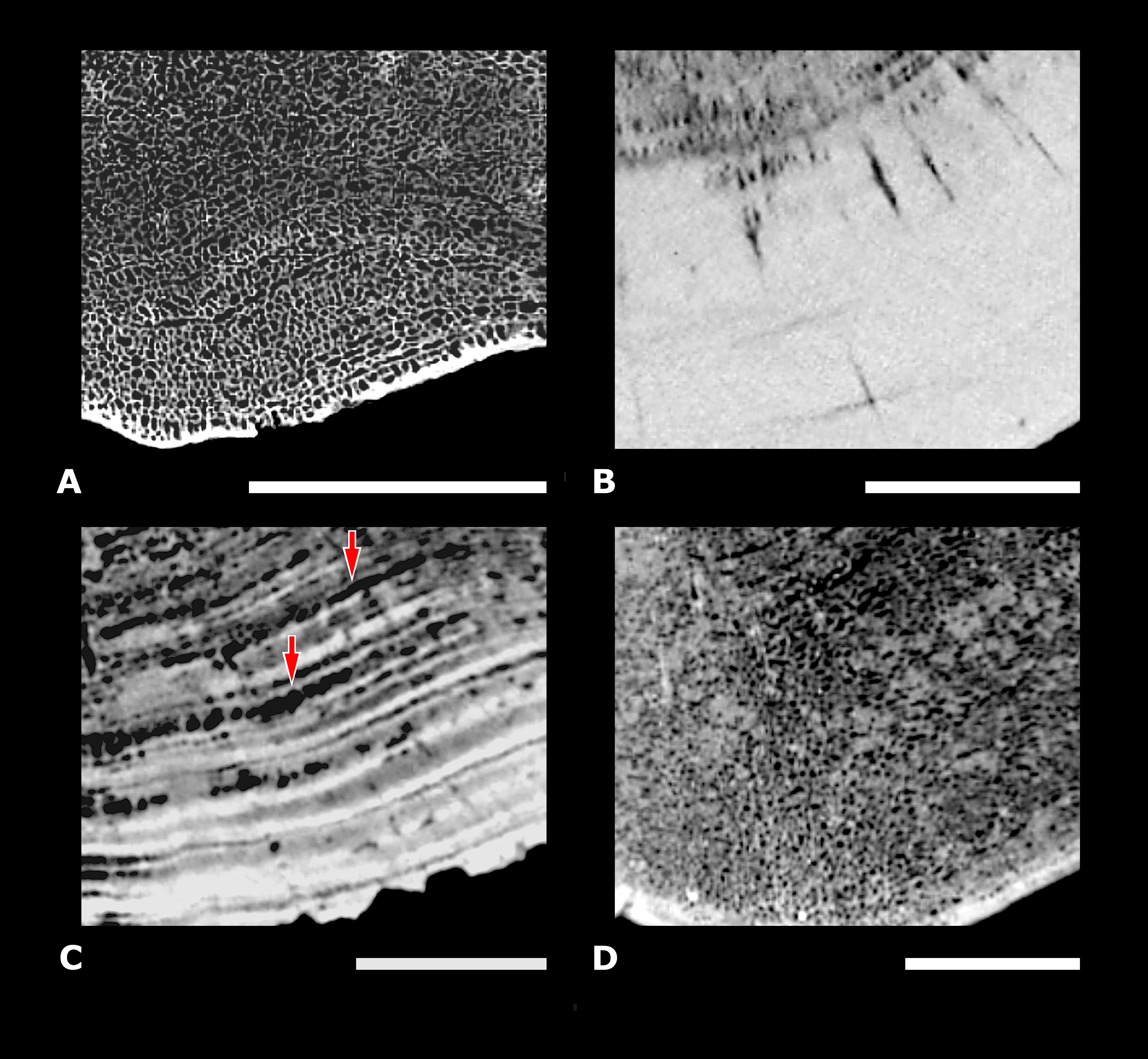

Supplement: Figure S4 — CT-scan images in axial view from the ventral part of the vertebral cortex in the morphotypes 1, 2 and 3. (A) Morphotype 1a, central-posterior thoracic vertebra, NMR-16642, small species of Pachycetus. (B) Morphotype 1b, central-posterior thoracic vertebra, sp., NMR-12331, large species of Pachycetus. (C) Morphotype 2, posterior thoracic or lumbar vertebra, NMR-10284, indeterminable basilosaurid. (D) Morphotype 3, caudal vertebra, NMR-10283, indeterminable basilosaurid. Vertical red arrows in (C) point to less compact layers, intercalated between compact layers in the cortex. See also Table S1. Scale bars are 25 mm. [file peerj-12-16541-s004.png]

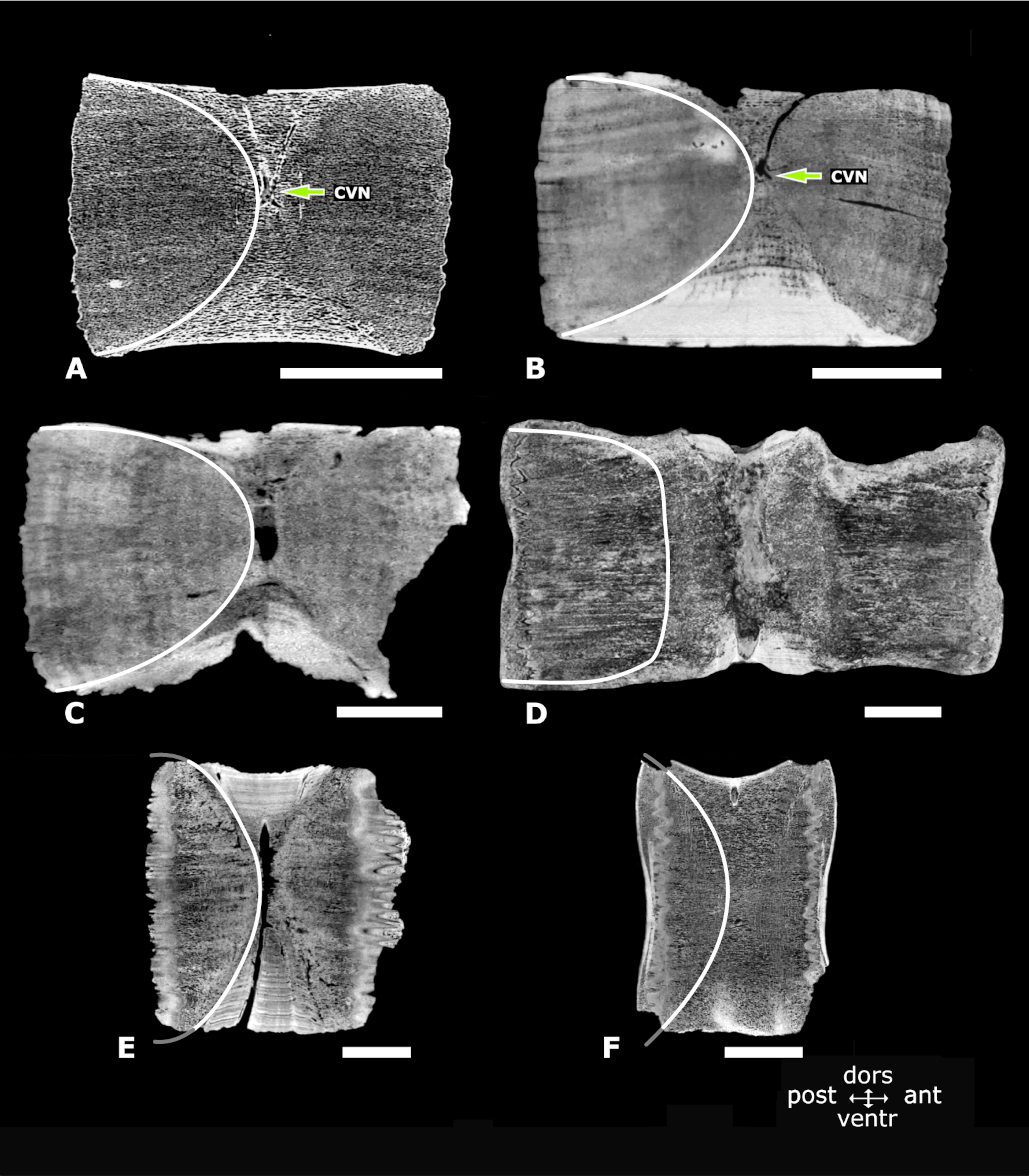

Supplement: Figure S5 — CT-scan images in sagittal view of sections at the midpart of vertebral centra in the morphotypes 1, 2 and 3 from Wielingen/Het Scheur at the Belgian-Dutch border, except (D), showing the shape of the cones. (A) Morphotype 1a, central-posterior thoracic vertebra, NMR-16642, small species of Pachycetus. (B) Morphotype 1b, central-posterior thoracic vertebra, NMR-12331, large species of Pachycetus. (C) Morphotype 1b, lumbar vertebra, large species of Pachycetus, NMR-3404. (D) Elongated lumbar or caudal vertebral centrum, USNM 510831, Basilosaurus cetoides, modified after (Houssaye et al. (2015): fig. 14a). (E) Morphotype 2, posterior thoracic or lumbar vertebra, NMR-10284, indeterminable basilosaurid. (F) Morphotype 3, caudal vertebra, NMR-10283, indeterminable basilosaurid. White lines indicate the shape of the cones. The grey lines outside the vertebra in (E) and (F) indicate the missing parts of the original cone. Green arrows in (A) and (B) point to the central vascular node (CVN). See also Suppl. Table S1. Scale bars are 50 mm. [file peerj-12-16541-s005.png]

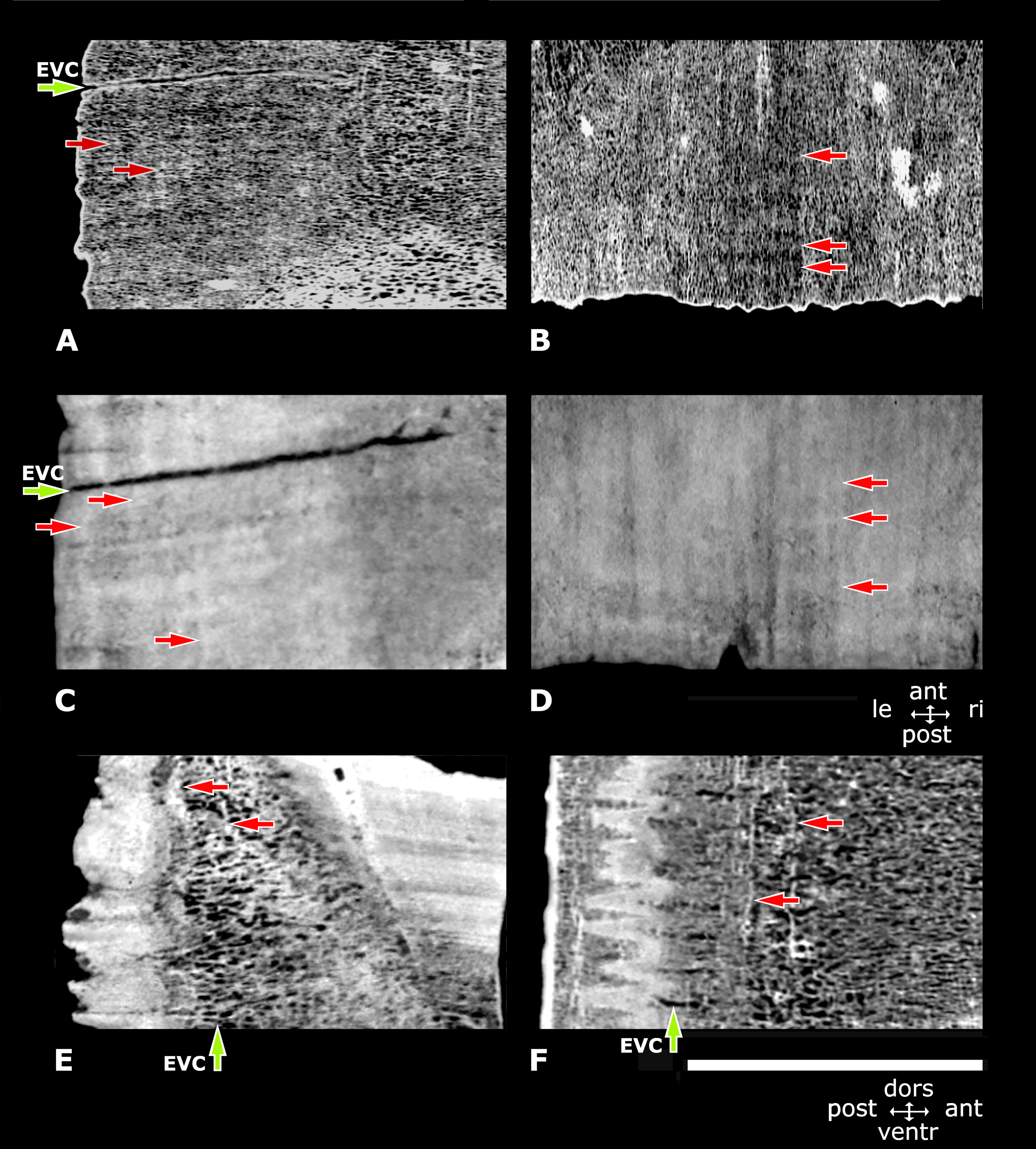

Supplement: Figure S6 — CT-scan images of the posterior cone in the morphotypes 1, 2 and 3. (A-B) Morphotype 1a, central-posterior thoracic vertebra, NMR-16642, small species of Pachycetus, in sagittal (A-B) and coronal (B) view. (C-D) Morphotype 1b, central-posterior thoracic vertebra, NMR-12331, large species of Pachycetus, sp., in sagittal (C) and coronal (D) view. (E) Morphotype 2, posterior thoracic or lumbar vertebra, NMR-10284, indeterminable basilosaurid in sagittal view. (F) Morphotype 3, caudal vertebra, NMR-10283, indeterminable basilosaurid in sagittal view. Red arrows in (A-F) point to the layering, seen in the cones, which indicates slower and more rapid growth. Green arrows (marked with ‘EVC’) in (A), (C), (E) and (F) point to endoconal vascular canals. In (A), (C), (E) and (F) left side is posterior; in (B) and (D) left side is left. See also Suppl. Table 1. Scale bars are 50 mm. [file peerj-12-16541-s006.png]

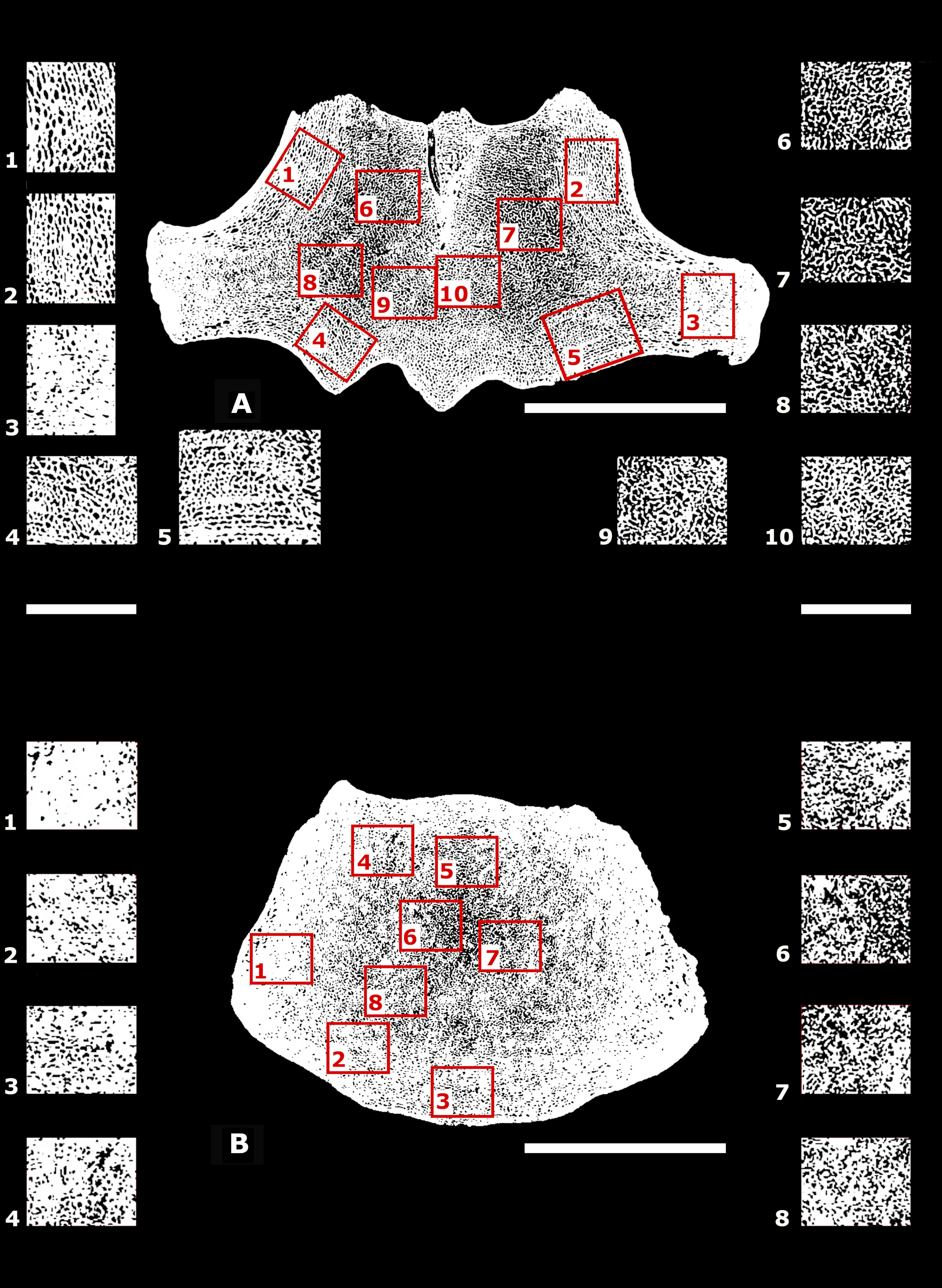

Supplement: Figure S7 — Sections of the vertebral centrum, NMR-16442, small species of Pachycetus, in axial view, used for measuring the bone compactness: (A) Vertebral midpart. (B) Anterior cone. See also Suppl. Table 1. Scale bars in (A) and (B) are 50 mm. Scale bars left and right below are 15 mm. [file peerj-12-16541-s007.png]

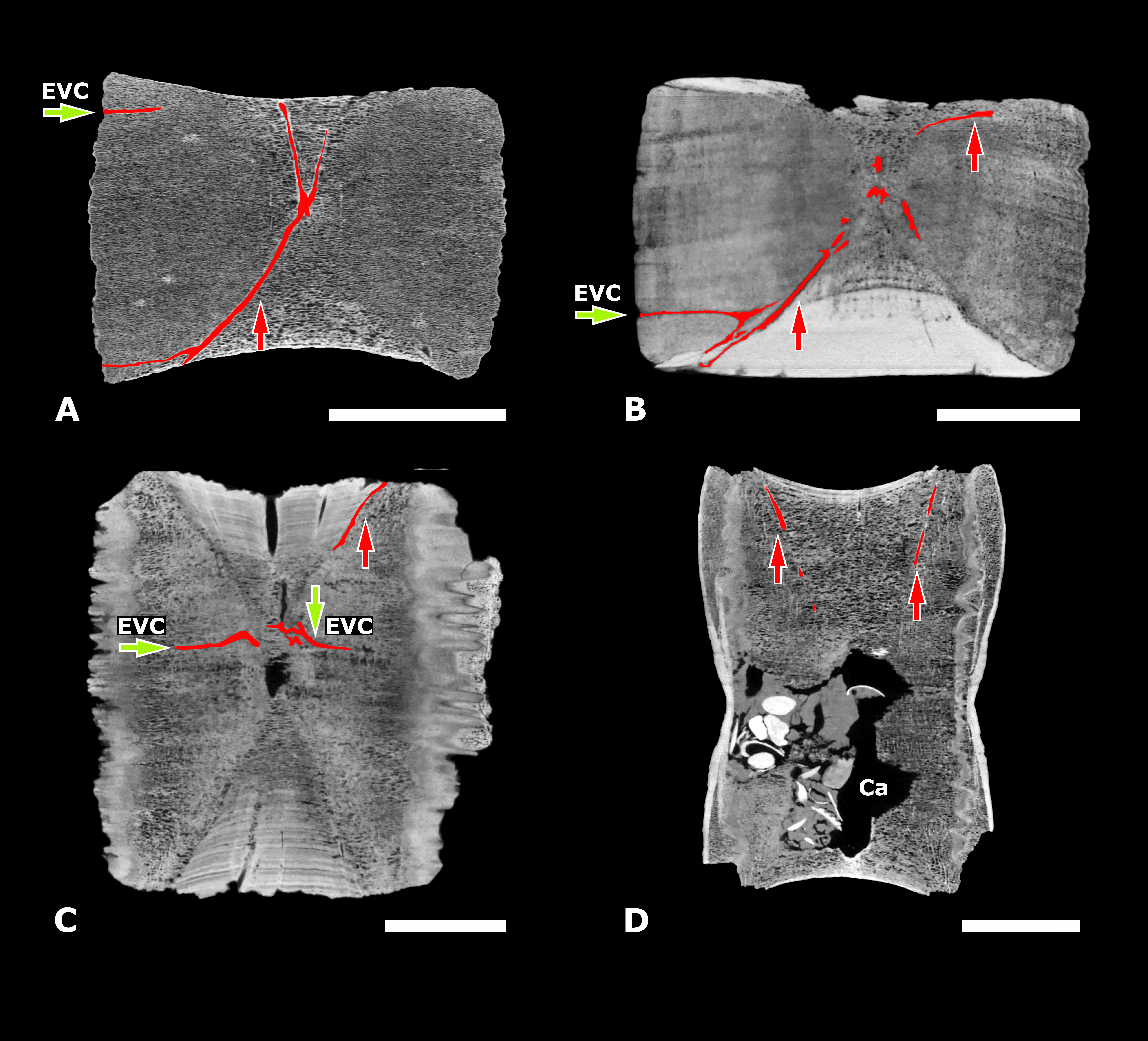

Supplement: Figure S8 — CT-scan images in sagittal view of the vertebral centra of the morphotypes 1, 2 and 3. (A) Morphotype 1a, central-posterior thoracic vertebra, NMR-16642, small species of Pachycetus. (B) Morphotype 1b, central-posterior thoracic vertebra, NMR-12331, large species of Pachycetus. (C) Morphotype 2, vertebral centrum, NMR-10284, indeterminable basilosaurid. (D) Morphotype 3, vertebral centrum, NMR-10283, indeterminable basilosaurid. The epiconal VC and some endoconal VC are coloured red. Red arrows point to an epiconal VC. Green arrows in (A), (B) and (C) point to an endoconal VC (EVC). Ca in (D) is a large cavity with infill of sediments and molluscs. See also Suppl. Table 1. Scale bars are 50 mm. [file peerj-12-16541-s008.png]

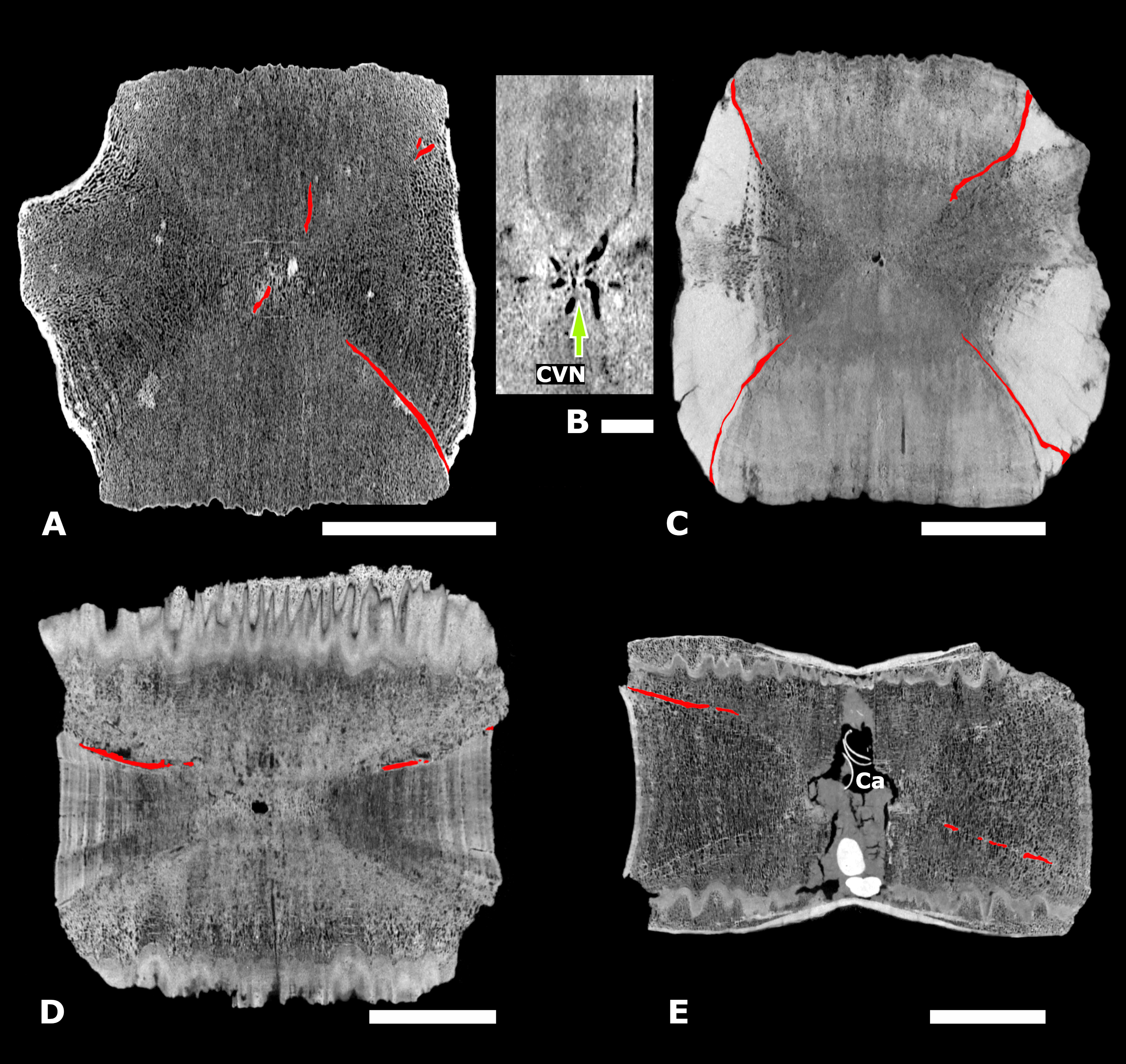

Supplement: Figure S9 — CT-scan images in coronal view of the vertebral centra of the morphotypes 1, 2 and 3, showing the epiconal VC. (A) Morphotype 1a, central-posterior thoracic vertebra, NMR-16642, small species of Pachycetus. (B-C) Morphotype 1b, central-posterior thoracic vertebra, NMR-12331, large species of Pachycetus. In (B) the confluence of epiconal canals in the central vascular node is seen. (D) Morphotype 2, vertebral centrum, NMR-10284, indeterminable basilosaurid. (E) Morphotype 3, vertebral centrum, NMR-10283, indeterminable basilosaurid. The epiconal VC are coloured red. Green arrow in (B) points to the central vascular node (CVN). Ca in (E) is a large cavity with infill of sediments and molluscs. See also Suppl. Table 1. Scale bars in (A), (C) (D) and (E) are 50 mm. Scale in (B) is 10 mm. [file peerj-12-16541-s009.png]

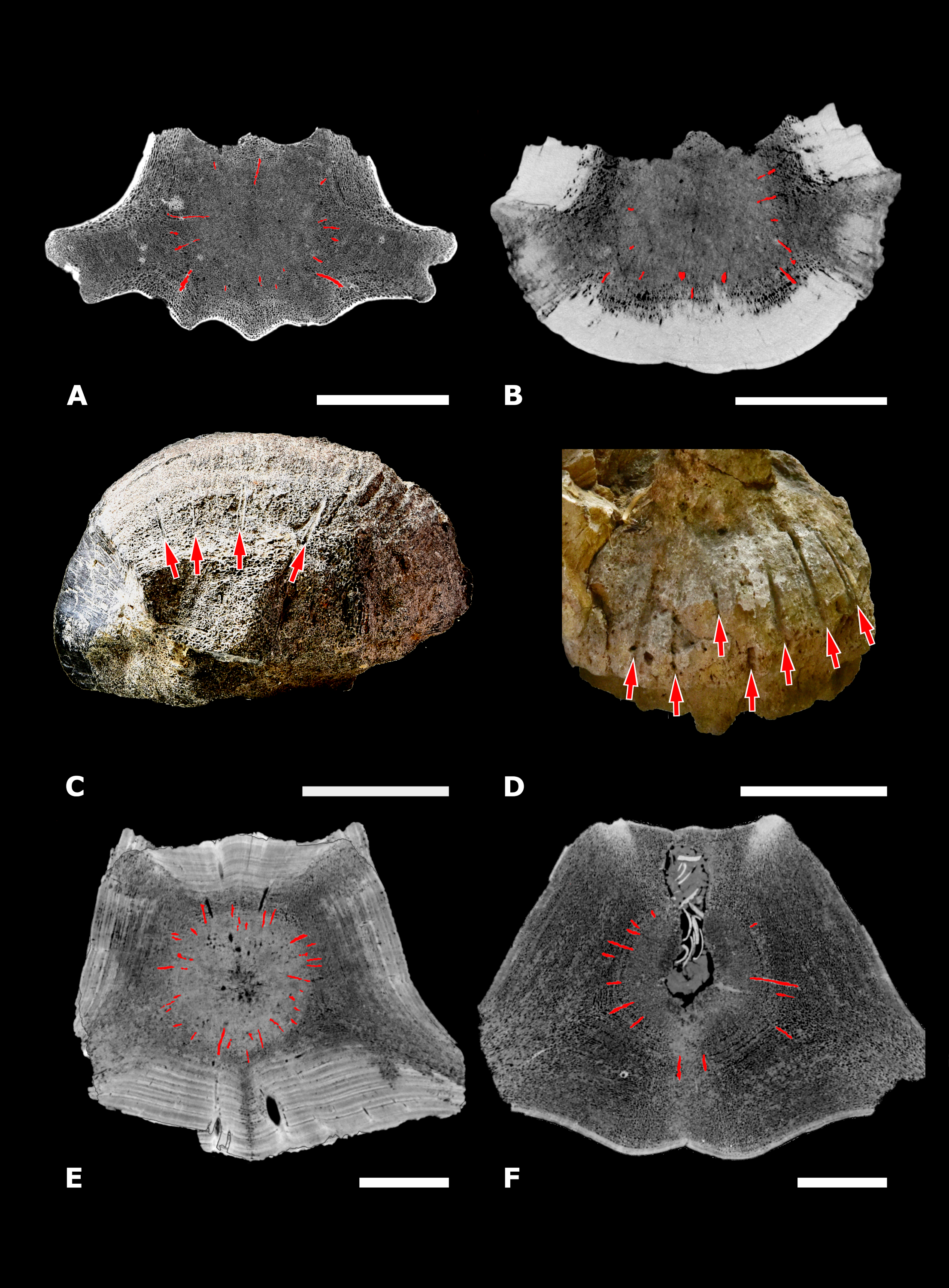

Supplement: Figure S10 — CT-scan images showing the epiconal VC in vertebrae of the morphotypes 1, 2 and 3, as well as two cones with imprints of epiconal vascular canals. (A) Morphotype 1a, vertebral centrum, NMR-16642, small species of Pachycetus in axial view.(B) Morphotype 1b, vertebral centrum, NMR-12331, large species of Pachycetus in axial view. (C) Morphotype 1b, partial anterior cone, ID20-4, large species of Pachycetus. (D) Morphotype 1b, cone, SMNS 10934b, large species of Pachycetus. (E) Morphotype 2, vertebral centrum, NMR-10284, indeterminable basilosaurid in axial view. (F) Morphotype 3, vertebral centrum, NMR-10283, indeterminable basilosaurid in axial view. The epiconal VC are coloured red. Red arrows in (C, D) point to some of the epiconal VC along the cones. See also Suppl. Table 1. Scale bar is 50 mm. [file peerj-12-16541-s010.png]

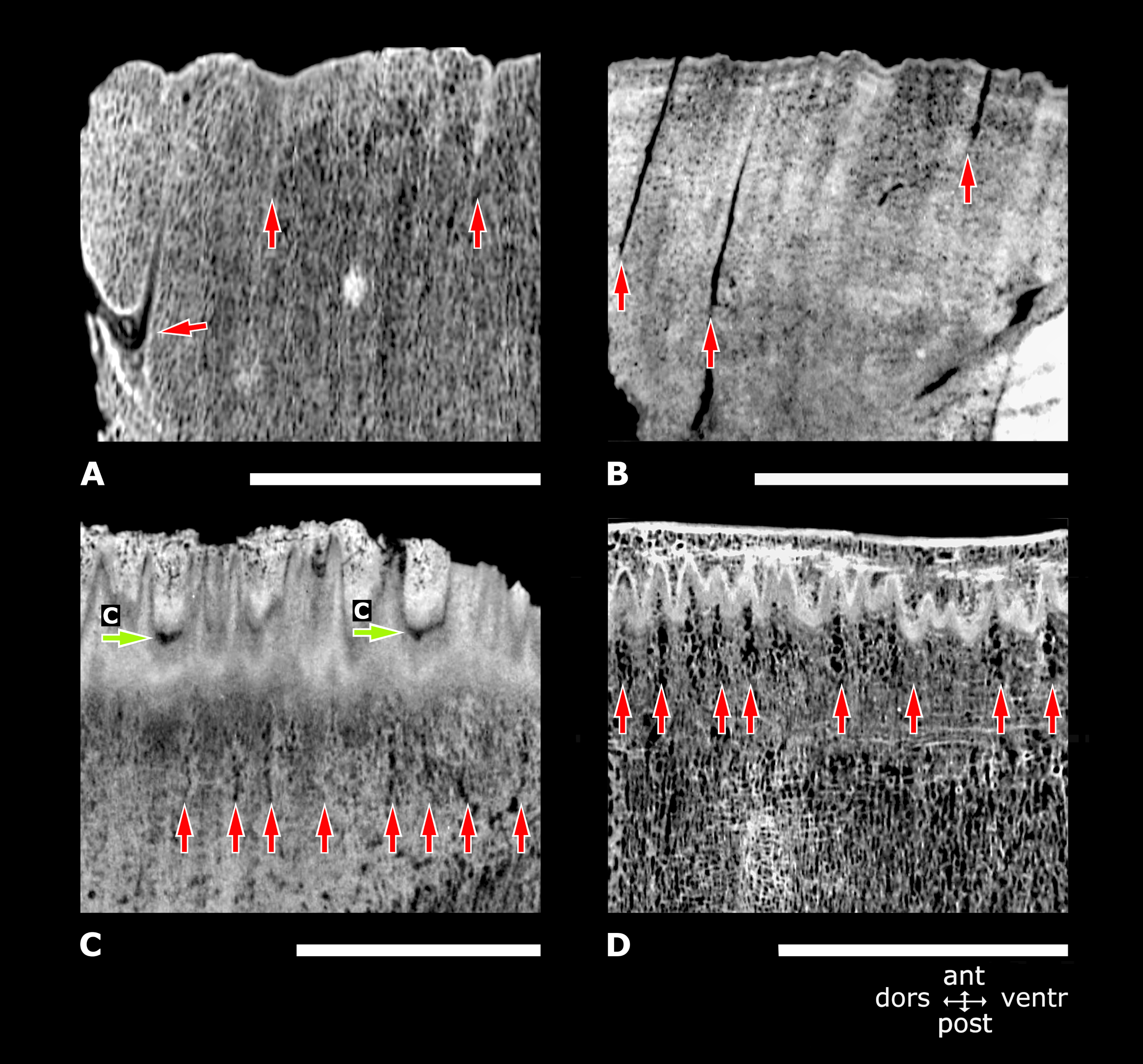

Supplement: Figure S11 — CT-scan images in sagittal view of the anterior cone in vertebrae of the morphotypes 1, 2 and 3. (A) Morphotype 1a, central-posterior thoracic vertebra, NMR-16642, small species of Pachycetus. The majority of the endoconal VC are hardly visible, but the branching of a large endoconal VC from an epiconal one is seen on the left. (B) Morphotype 1b, central-posterior thoracic vertebra, NMR-12331, large species of Pachycetus. (C) Morphotype 2, posterior thoracic or lumbar vertebra, NMR-10284, indeterminable basilosaurid. (D) Morphotype 3, caudal vertebra, NMR-10283, indeterminable basilosaurid. Red arrows point to the endoconal VC, which end between the ridges of the epiphyseal side in (D). Horizontal green arrows (marked with ‘C’) in (C) point to capillaries or maybe places where cartilage had been. See also Suppl. Table S1. Scale bars are 50 mm. [file peerj-12-16541-s011.png]

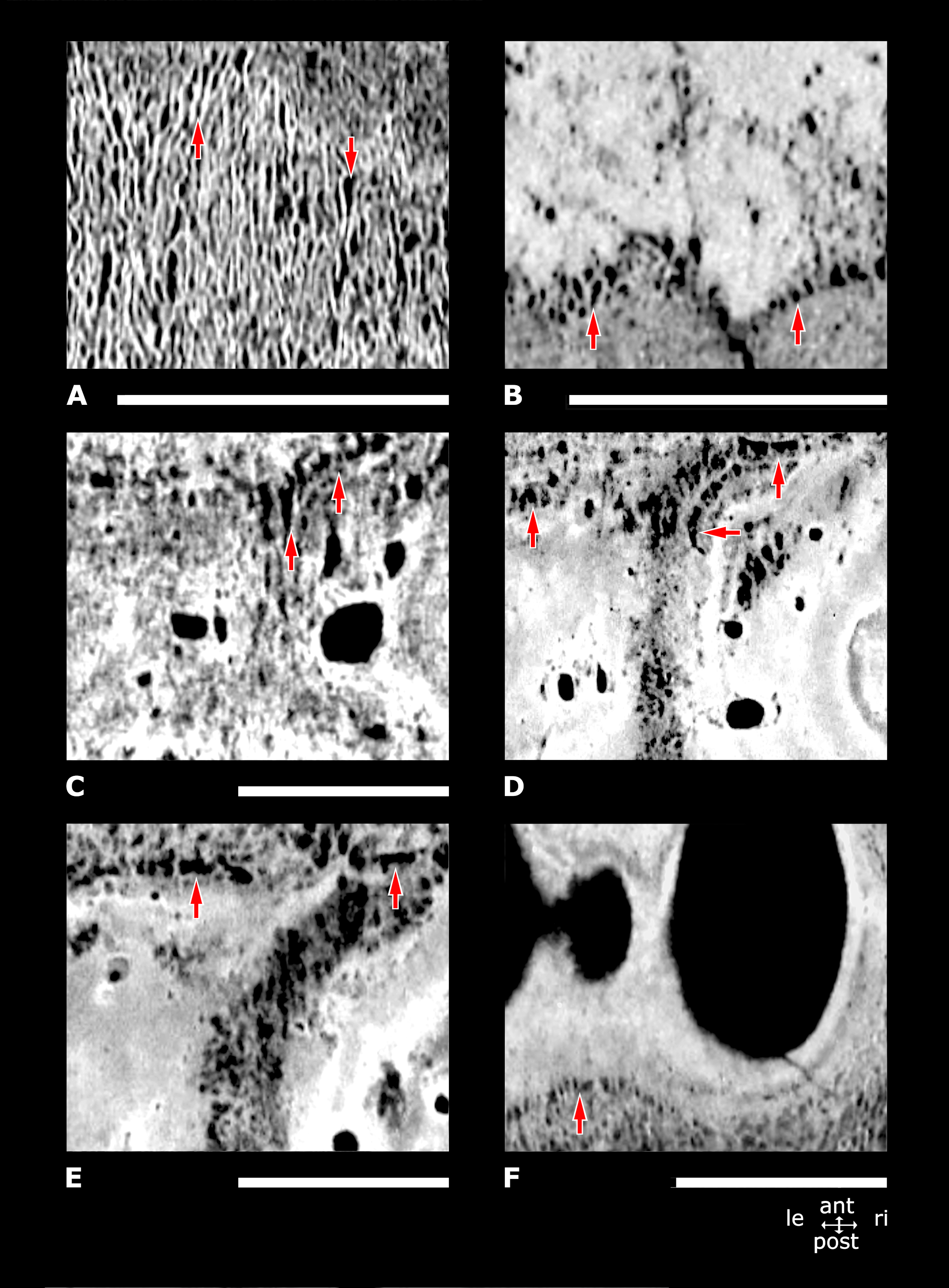

Supplement: Figure S12 — CT-scan images in coronal view of the midpart of the ventral cortex in vertebral centra of the morphotypes 1, 2 and 3, showing the layers with endocortical VC, present in vertebrae with a loose or a compact cortex. (A) Morphotype 1a, central-posterior thoracic vertebra, NMR-16642, small species of Pachycetus. (B) Morphotype 1b, central-posterior vertebra, NMR-12331, large species of Pachycetus. (C-E) Morphotype 2, posterior thoracic or lumbar vertebra, NMR-10284, indeterminable basilosaurid. C to E show the succession of layers with endocortical VC from the cortex toward the ventral surface. (F) Morphotype 3, caudal vertebra, NMR-10283, indeterminable basilosaurid (with a very large and a smaller ventral foramen). The cavities are interpreted as vascular canals, because of their arrangement in well-ordered, circular lines and their shape is much alike, contrary to trabecular spaces, with are irregular and unevenly distributed. Red arrows point to the anterior-posterior running endocortical VC in layers of the inner cortex. See also Table S1. Scale bars are 25 mm. [file peerj-12-16541-s012.png]

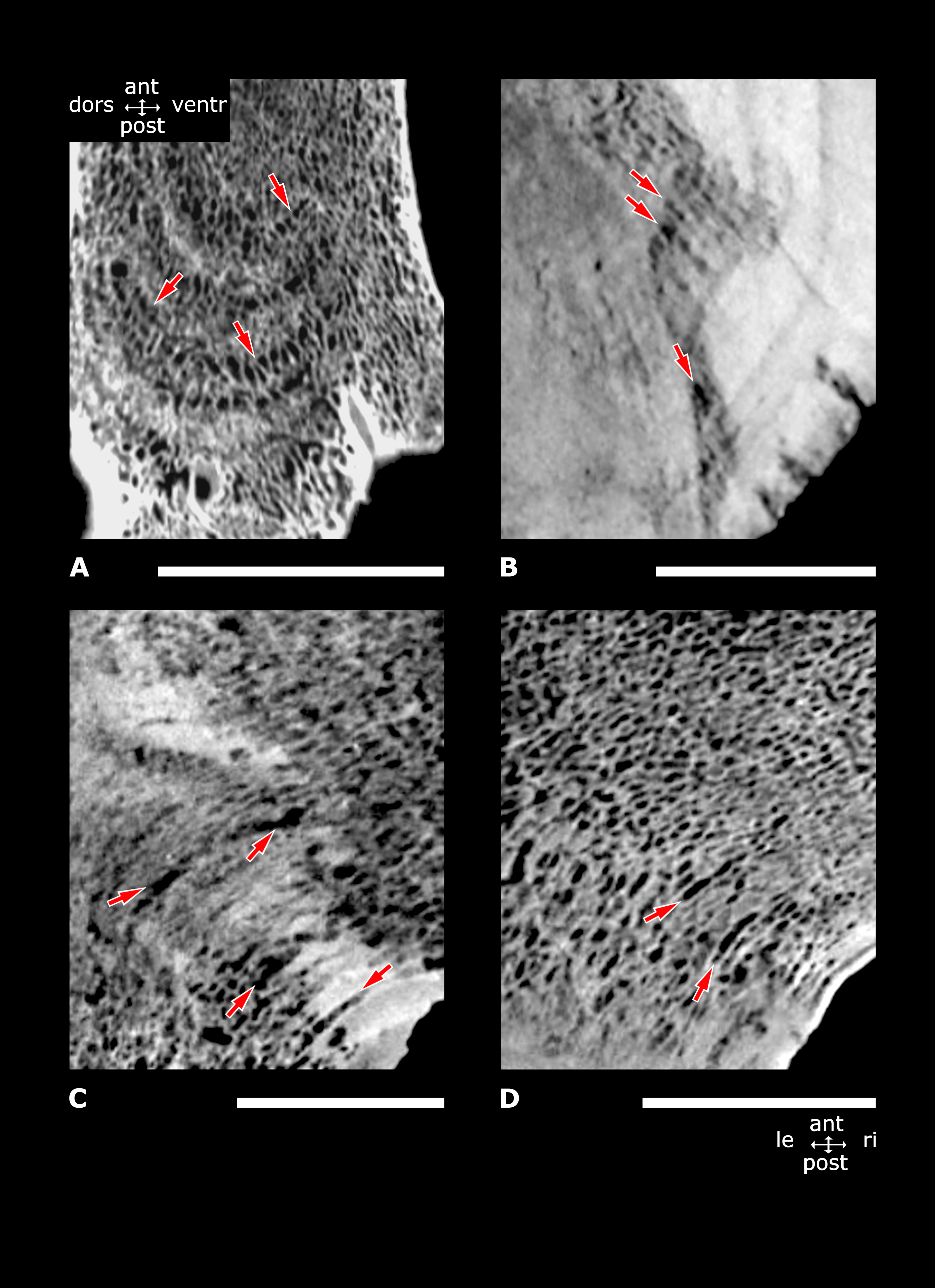

Supplement: Figure S13 — CT scan images of the cortex in the transverse processes in vertebral centra of the morphotypes 1, 2 and 3, showing the layers with endocortical VC in the transverse processes, present in vertebrae with a loose or a compact cortex. (A) Morphotype 1a, central-posterior thoracic vertebra, NMR-16642, small species of Pachycetus in sagittal view. (B) Morphotype 1b, central-posterior thoracic vertebra, NMR-12331, large species of Pachycetus in coronal view. (C) Morphotype 2, posterior thoracic or lumbar vertebra, NMR-10284, indeterminable basilosaurid in coronal view. (D) Morphotype 3, caudal vertebra, NMR-10283, indeterminable basilosaurid in coronal view. The cavities are interpreted as vascular canals, because of their arrangement in well-ordered, circular lines and their shape is much alike, contrary to trabecular spaces, with are irregular and unevenly distributed. Red arrows point to the directions of the endocortical VC in layers of the inner cortex, running toward the end of the transverse process. In (A), left side is dorsal; in (B), (C) and (D), left side is left. See also Table S1. Scale bars are 50 mm. [file peerj-12-16541-s013.png]

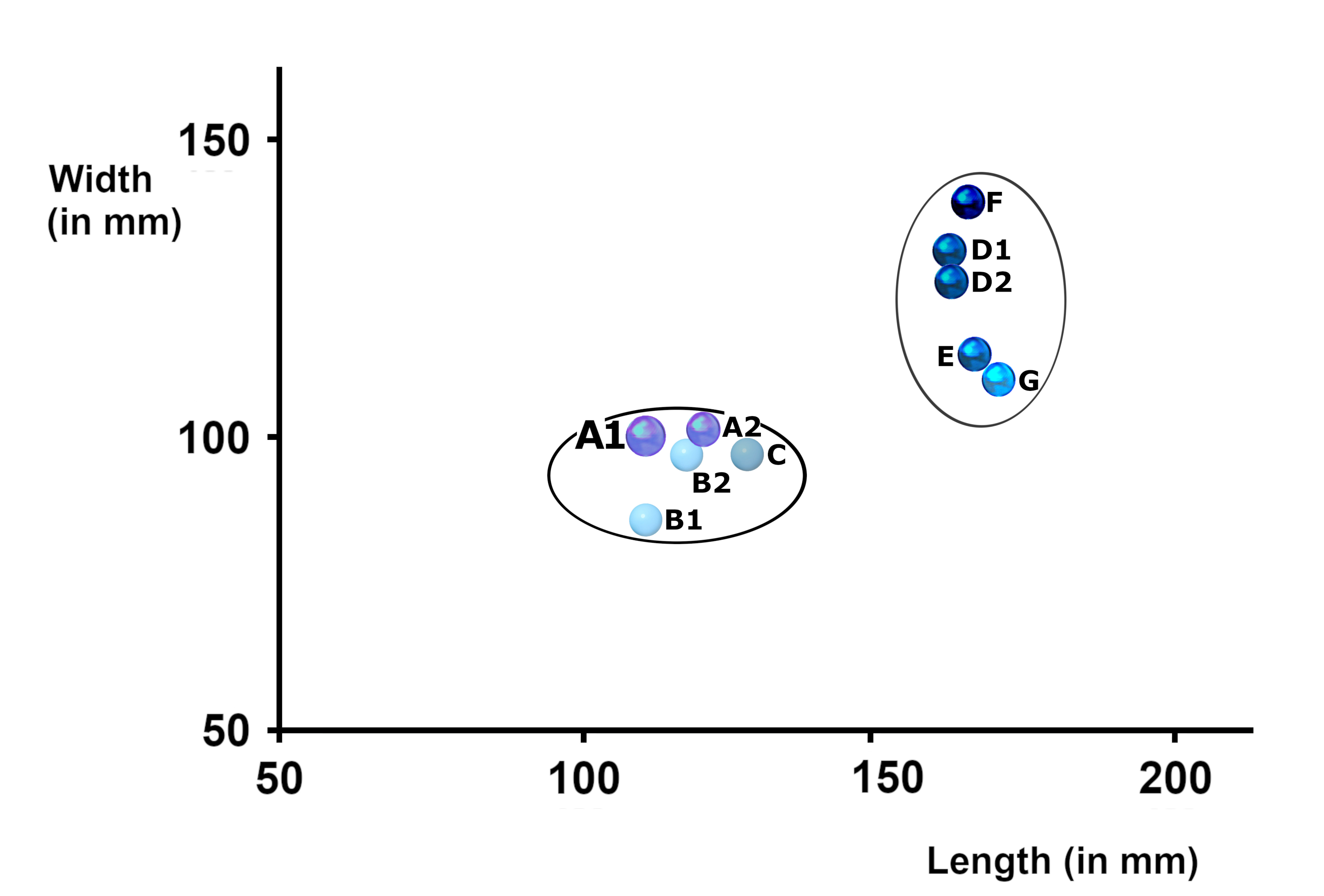

Supplement: Figure S14 — Scatter diagram with width plotted against length of central-posterior thoracic vertebral centra, all assigned to Pachycetus spp. (NMR150839 is a posterior thoracic or maybe a lumbar vertebra). Two different clusters of vertebrae are seen: the spheres ‘A’-‘C’ within the ellipsis to the left, represent vertebrae belonging to small species of Pachycetus, including vertebrae NMR-16642 and NMR150839 from Het Scheur at the Belgian-Dutch border. The spheres ‘D’-‘G’ within the ellipsis to the right, represent vertebrae, belonging to large species of Pachycetus. (A1-2) NMR-16642, resp. NMR-150839 (posterior thoracic/lumbar vertebra) from Het Scheur at the Belgian-Dutch border; (B1-2) Pachycetus wardii, NCSM 11284 from North Carolina; (C): Antaecetus aithai, FSAC Bouj-7 from Guéran, Morocco; (D2) Pachycetus sp., NMR-12331 and (D1) NMR-12332 from Het Scheur at the Belgian-Dutch border; (E) P. robustus, NsT90, from the Helmstedt Region, Germany, Belgium; (F) P. paulsonii; from Chyhyryn, Ukraine; (G) Basilotritus (Pachycetus) uheni, 44761 P 203 from Vlavovska, Ukraine. See also Table S1. [file peerj-12-16541-s014.png]

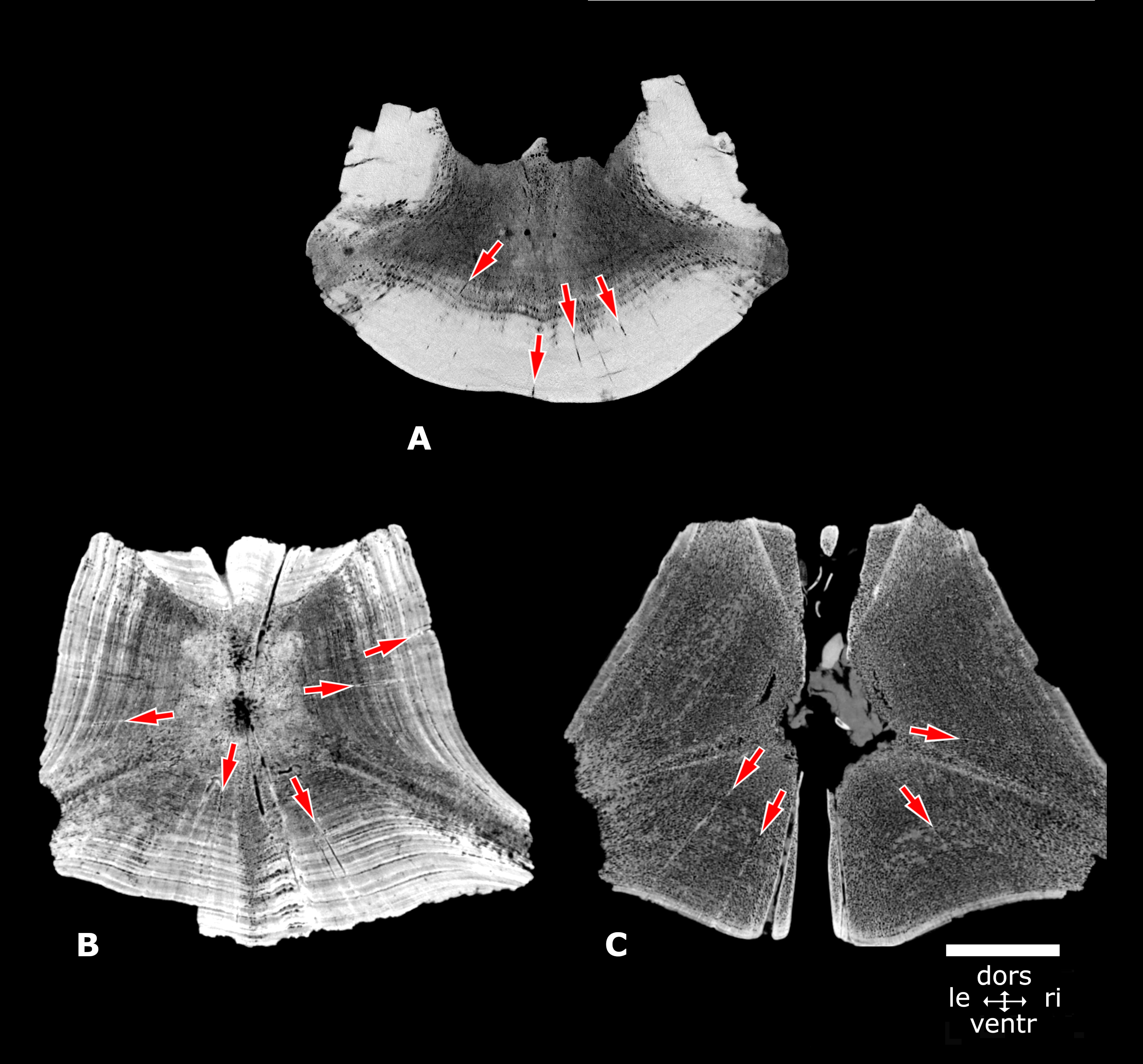

Supplement: Figure S15 — CT-scan images in axial view of vertebrae of morphotypes 1b, 2 and 3, showing the accessory VC. (A) Morphotype 1b, central-posterior thoracic vertebra, NMR-12331, large species of Pachycetus. (B) Morphotype 2, posterior thoracic or lumbar vertebra, NMR-10284, indeterminable basilosaurid. (C) Morphotype 3, caudal vertebra, NMR-10283, indeterminable basilosaurid. The accessory VC can be discerned, because they are running in a straight line from the central vascular node toward the vertebra’s surface. Red arrows point to the tiny accessory VC. See also Table S1. Scale bar is 50 mm. [file peerj-12-16541-s015.png]
