# Supplementary material for: A vertebra of a small species of Pachycetus from the North Sea and its inner structure and vascularity compared with other basilosaurid vertebrae from the same site
Source: PeerJ. 2024 Jan 25;12:e16541. doi: 10.7717/peerj.16541 (PMC11107809; doi:10.7717/peerj.16541)
Supplement: Table S1 [file peerj-12-16541-s016.docx]

**SUPPL. TABLE 1: Vertebrae mentioned in the Figures**

| Collection number & taxon | Figures & Suppl.  Figures | Vertebra | Country & age | Length | Width | Height | Rel L=  Ld/Wa | References |
| --- | --- | --- | --- | --- | --- | --- | --- | --- |
| **Type 1a: elongated vertebrae (small)** |  |  |  |  |  |  |  |  |
| NMR-16642  *Pachycetus* sp., small species | Figs 3; 5,6A-D; 7B-C; 8A-B; 9A-B; 10A  Suppl. Figs 1(1a**^1^)**; 3A; 4A; 5A; 6A-B; 7; 8A; 9A; 10A; 11A; 12A; 13A, 14(A**^1^**) | Central-post.Th**^**^**)  (Th8-11) | Het Scheur, Belgian-Dutch border;  Bartonian | 111 (dors)  110 (vent) | 101 (ant)  102 (post) | 80 (ant)  83.5 (post) | 1.1 | This article |
| NMR-150839  *Pachycetus* sp., small species | Fig. 9C-D  Suppl. Figs 1(1a**^2^)**, 14(A**^2^)** | Post Th/Lu | Het Scheur, Belgian-Dutch border;  Bartonian | 121 (dors)  118 (vent) | 102 (ant?)  105 (post?) | 89 (ant?)  82 (post?) | 1.19 | Van Vliet et al., 2022 |
| MGSB25.191  *Pachycetus* sp., small species | Fig. 10B | Ca | Taradell, Spain; Bartonian? | 110  e138 | >110 (ant)  112 (post) | >77 (ant)  104 (post) | e1.23 | Van Vliet et al., 2023 |
| NCSM 11284.  *Pachycetus wardii* | Suppl. Fig. 14B**^1^** | Th11*) | North Carolina; Lutetian-Bartonian | ? (dors)  111 (ventr) | 86 (ant)  109 (post) | 73 (ant)  81 (post) | 1.29 (Lv) | Uhen, 2001: table 1 |
| NCSM 11284.  *Pachycetus wardii* | Suppl. Fig. 14B**^2^** | Th12**) | North Carolina; Lutetian-Bartonian | ? (dors)  118 (ventr) | 97 (ant)  112 (post) | 82 (ant)  86 (post) | 1.22 (Lv) | Uhen, 2001: table 1 |
| FSAC Bouj-7  *Antaecetus aithai* | Suppl. Fig. 14C | Th12 | Guéran, Morocco,  Bartonian | 128 | e97 (ant)  96 (post) | e82 (ant’  77 (post) | e1.32 | Gingerich & Zouhri, 2015: table 2 |
| **Type 1b: elongated vertebrae (large)** |  |  |  |  |  |  |  |  |
| NMR-12332  *Pachycetus* sp.,  large species | Figs 6I-L; 7A, C; 8E  Suppl. Figs 1(1b**^1^),** 14D**^1^** | Central-post. Th**^**^**)  (Th7-10) | Het Scheur, Belgian-Dutch border;  Bartonian | 162 (dors)  155 (vent) | 132 (ant)  152 (post) | 109 (ant)  114 (post) | 1.23 | Van Vliet et al., 2022 |
| NMR-12331  *Pachycetus* sp., large species | Figs 6E-H  Suppl. Figs 1(1b**^2^);** 3B; 4B; 5B; 6C-D; 8B; 9B-C; 10B; 11B; 12B; 13B; 14D**^2^**; 15A | Central-post. Th**^**^**)  (Th7-10) | Het Scheur, Belgian-Dutch border;  Bartonian | 163 (dors)  158 (vent) | 127 (ant)  147 (post) | 107 (ant)  102 (post) | 1.28 | Van Vliet et al., 2022 |
|  |  |  |  |  |  |  |  |  |
| NMR-16645  *Pachycetus* sp., large species | Suppl. figure 1(1b**^3^)** | Lu**) | Het Scheur, Belgian-Dutch border;  Bartonian | 204 (dors)  195 (vent) | e148 (ant)  156 (post) | e120 (ant)  124 (post) | e1,38 | Van Vliet et al., 2022 |
| NMR-3404  *Pachycetus* sp., large species | Fig. 6M-P  Suppl. figure 1(1b**^4^)**; 3C; 5C | Lu**) | Het Scheur, Belgian-Dutch border;  Bartonian | >208 (dors)  >165 (vent) | >150 (ant)  e175 (post) | e125 (ant)  124 (post) | ? | Van Vliet et al., 2022 |
| NsT90  *Pachycetus robustus* | Fig. 8C-D  Suppl. Fig. 14E | Post. Th  (?ant Lu) **^**^**) | Helmstedt region, Germany; Bartonian-Priabonian | 166 (dors)  162 (vent) | >114 (ant)  129 (post) | >81 (ant)  96 (post) | <1.46  (>1.28)  (Ld/Wp) | Van Beneden, 1883;  Kuhn, 1935;  Van Vliet et al., 2020: appendix table 2a |
| *Pachycetus paulsonii* | Suppl. Fig. 14F | Central Th | Chyhyryn, Ukraine; Bartonian-Priabonian | 165 | 140 | 100 | 1.18 | Brandt, 1873;  Kellogg, 1936: table 25 |
| KOM 44761 P 203 *Basilotritus* (*Pachycetusi*) *uheni* | Suppl. Fig. 14G | ThB (12 or 13) **) | Vlavoska, Ukraine  Bartonian-Priabonian | 170 | 110 (ant)  149 (post) | 96 (ant)  108 (post) | 1.55 | Gol’din & Zvonok, 2013, appendix 3 |
|  |  |  |  |  |  |  |  |  |
| ID20-4  *Pachycetus* sp. | Suppl. Fig. 10C | Conus, Lu | Helmstedt region, Germany; Bartonian-Priabonian | ? | ? | ? | ? | Van Vliet et al., 2022: plate 3B1 |
| SMNS 10934b,  *Pachycetus* sp. | Suppl. Fig. 10D | Conus, Lu? | Mokattam, Cario Egypt; Bartonian | ? | ? | ? | ? | Stromer, 1908;  Gingerich et al., 2022 |
| USNM 510831  *Basilosaurus cetoides* | Suppl. Figs 3D; 5D | Lu/Ca | ? | ? | ? | ? | ? | Houssaye et al., 2015: fig. 14 |
| **Type 2: not-elongated vertebrae** |  |  |  |  |  |  |  |  |
| NMR-10284  Indeterminable basilosaurid | Suppl. Figs 1(2); 3E; 4C; 5E; 6E; 8C; 9D; 10E; 11C; 12C-E; 13C; 15B | Th/Lu**^**^**) | Wielingen/ Het Scheur, Belgian-Dutch border;  Bartonian-Priabonian | 177 (dors)  170 (vent) | >190 (ant)  e>187 (post) | > 180 (ant)  > 183 (post) | < 0.93 | Van Vliet et al., 2022 |
| **Type 3: ‘shortened’ vertebrae** |  |  |  |  |  |  |  |  |
| NMR-160635 | Suppl. Fig. 1(3**^1^**) | ?Th1?**^**^**) | Wielingen/ Het Scheur, Belgian-Dutch border;  Bartonian-Priabonian | 56 (dors)  57 (vent) | 103 (ant  126 (post) | 75 (ant)  82 (post) | 0,54 | Van Vliet et al., 2022 |
| NMR-3882) | Suppl. Fig. 1(3**^2)^** | Lu**^**^**) | Wielingen/ Het Scheur, Belgian-Dutch border;  Bartonian-Priabonian | 94 (dors)  92 (vent) | 178 (ant)  180 (post) | 145 (ant)  145 (post) | 0,53 | Van Vliet et al., 2022 |
| NMR-3403 | Suppl. Fig. 1(3**^3^**) | Lu**^**^**) | Wielingen/ Het Scheur, Belgian-Dutch border;  Bartonian-Priabonian | 110 (dors)  112 (vent) | 229 (ant)  228 (post) | 183 (ant)  190 (post) | 0,48 | Van Vliet et al., 2022 |
| NMR-3402 | Suppl. Fig. 1(3**^4^**) | Post. Th**^**^**) | Wielingen/ Het Scheur, Belgian-Dutch border;  Bartonian-Priabonian | 123 (dors)  118 (vent) | 237 (ant)  231 (post) | 179 (ant)  180 (post) | 0,52 | Van Vliet et al., 2022 |
| NMR-10283  Indeterminable basilosaurid | Suppl. Figs 1(3**^5^**); 3F; 4D; 5F; 6F; 8D; 9E; 10F; 11D; 12F; 13D; 15C. | Ca | Wielingen/ Het Scheur, Belgian-Dutch border;  Bartonian-Priabonian | 127 (dors)  135 (vent) | 210 (ant)  212 (post) | 191 (ant)  185 (post) | 0.6 | Van Vliet et al., 2022 |
|  |  |  |  |  |  |  |  |  |

**^*^**), One epiphysis missing; **^**^**), both epiphyses missing

*Abbreviations:*

ant, anterior; Ca, caudal vertebral centrum; dors, dorsal; e, estimated; et al., et alii; Ld, dorsal length; Lu, lumbar vertebral centrum; Lv, ventral length; post, posterior; rel, relative; Th, thoracic vertebral centrum; sp, species; vent, ventral; Wa, anterior width; Wp, posterior width;?, unknown; >, more than; <, less than; /, fraction
